# China's Rural Public Health System Performance: A Cross-Sectional Study

**DOI:** 10.1371/journal.pone.0083822

**Published:** 2013-12-26

**Authors:** Miaomiao Tian, Da Feng, Xi Chen, Yingchun Chen, Xi Sun, Yuanxi Xiang, Fang Yuan, Zhanchun Feng

**Affiliations:** 1 School of Medicine and Health Management, Huazhong University of Science and Technology, Wuhan, Hubei, China; 2 Institute of Medical Information, Center for Health Policy and Management, Chinese Academy of Medical Sciences, Beijing, China; 3 School of Management, Jiangsu University, Zhanjiang, Jiangsu, China; Iran University of Medical Sciences, Iran (Islamic Republic of)

## Abstract

**Background:**

In the past three years, the Government of China initiated health reform with rural public health system construction to achieve equal access to public health services for rural residents. The study assessed trends of public health services accessibility in rural China from 2008 to 2010, as well as the current situation about the China's rural public health system performance.

**Methods:**

The data were collected from a cross-sectional survey conducted in 2011, which used a multistage stratified random sampling method to select 12 counties and 118 villages from China. Three sets of indicators were chosen to measure the trends in access to coverage, equality and effectiveness of rural public health services. Data were disaggregated by provinces and by participants: hypertension patients, children, elderly and women. We examined the changes in equality across and within region.

**Results:**

China's rural public health system did well in safe drinking water, children vaccinations and women hospital delivery. But more hypertension patients with low income could not receive regular healthcare from primary health institutions than those with middle and high income. In 2010, hypertension treatment rate of Qinghai in Western China was just 53.22% which was much lower than that of Zhejiang in Eastern China (97.27%). Meanwhile, low performance was showed in effectiveness of rural public health services. The rate of effective treatment for controlling their blood pressure within normal range was just 39.7%.

**Conclusions:**

The implementation of health reform since 2009 has led the public health development towards the right direction. Physical access to public health services had increased from 2008 to 2010. But, inter- and intra-regional inequalities in public health system coverage still exist. Strategies to improve the quality and equality of public health services in rural China need to be considered.

## Introduction

China's health system has experienced remarkable changes over the past few decades since the market-oriented reforms launched in 1980s, which led China into a rapid economic growth period with dramatic social and political transitions. [1] The dramatic socioeconomic transitions have had significant impacts on health: overall, people in China live longer and healthier, their average life expectancy increased from 67.9 years in 1981 to 73.5 years in 2010. [2] From 1990 to 2010, the infant mortality rate (per 1,000 live births) decreased from 50.2 to 13.1, and the maternal mortality rate (per 100,000 live births) fell from 88.9 to 30.0. [3], [4] However, the performance of urban health system and rural health system did not improve at the same pace, with urban areas on balance prospering far more than rural ones. [5], [6] In 2008, the infant mortality rate in urban areas was 6.6 (per 1,000 live births), whereas it was up to 18.4 in rural areas. Moreover, there were 6.68 health professionals per 1,000 population in urban areas, but the rate in rural areas was only less than 3 (2.8 in 2008) [7]. Growing inequalities in the accessibility of public health services and health outcomes between rural and urban areas are the key problems that the China Government has to face, and the weakest link of public health is still in rural areas. [8]–[10]

Public health is the collective action for sustained population-wide health improvement which emphasizes sustainability of the practice (i.e., the need to embed policies within supportive systems) and reducing health inequalities. [11] In April 2009, China implemented her ambitious healthcare reform plan and funded RMB 850 billion (US$130·77 billion, RMB 6.5 per 1 US$) in the ensuing 3 years. [12] Two of the forced reforms are for improving the primary care delivery system so as to provide essential health care and making public health services available and equal for all. [13] In July 2009, nine essential public health services were carried out for all residents in China. [14] Meanwhile, the funding standard for essential public health services was increased from RMB 15 per capita (the average per capita public health funding) in 2009 to RMB 25 per capita in 2011. In 2009, nearly 60% of the total funding for essential public health services (RMB 10.4 billion) was used for rural residents. [15] Rural public health has become one of the highest priority areas of China's health system, more and more attention has been paid on improving rural residents' health and equalized the public health services. However, along with the policy actions implemented in rural areas, crucial questions can be raised: how has the China's rural public health system performance been? Will the China's health reform improve the rural public health system performance? How to assess the rural public health system performance?

In China, rural public health system consists two parts: on the one hand is the institutions of providing public health services, including county hospitals, centers for disease control and prevention, county emergency center, township hospitals and village clinics; on the other hand is the management and cooperation institutions, including health bureau, education bureau, bureau of quality and technical supervision, food and drug administration, blood banks, water companies, schools as well as local governments. The performance of public health system depends on the combined effects of the two components. Therefore, the assessment of public health system in this study focuses on the direct and indirect effects on performance from the whole rural public health system.

Although each province in China has its own performance evaluation plan on the essential public health services which focus on the public health staff's working performance [16], few researchers (Yip et al, 2012) have focused on the China's rural public health system performance and no data have been reported (published) for the equitable distribution of public health services. [17] Researches (Mays et al, 2006; Scutchfield et al, 2009) of public health performance in U emphasize the national measurement for public health system is effective to monitor the progress, identify the determinants of achieving goals, and provide performance data for policy planning. [18], [19] This study aimed at providing the up-to-date evidence and a performance-assessment toolbox for assessing the trends of public health services accessibility and financial protection in rural China from 2008 to 2010, as well as the current situation about the China's rural public health system performance.

## Methods

### Ethics statement

All of the research methods and investigational tools in this study were approved by the Ethics Committee of Tongji Medical College, Huazhong University of Science and Technology (IORG No: IORG0003571). All of the respondents and children's guardians in this manuscript gave a written informed consent to participate in the study, provided consent before filling out the questionnaire, and consented to the publication of the data.

### Performance-assessment toolbox

Performance measurement of public health system has been a popular research topic among the Centers for Disease Control and Prevention of the US, World Bank, researchers of Canada, and other researchers and organizations in the past 12 years. [20]–[22] In 2001, American researchers developed a conceptual framework to measure the performance of public health system, which was based on the instruments for assessing the performance of local and state public health departments developed by the Centers for Disease Control and Prevention's National Public Health Performance Standards Program. In the conceptual framework, resources, essential public health services, effectiveness, efficiency and equality of public health services are the five crucial components to assess public health system performance, which are also the goals of public health systems. [23] After considering the goals of rural public health system and the conceptual framework, three fundamental measures were involved in the performance-assessment toolbox, which were: 1) rural public health system coverage; 2) equality of rural public health services; and 3) effectiveness of rural public health services. In this paper, the rural public health system is referred to a three-tiered public health system within the administrative areas of county, township and village in China with the defining goals to improve rural residents' health sustainability and achieve the equity of essential public health services.

First, the measurement of rural public health system coverage focused on assessing the performance of public health care delivery and health resource allocation (e.g. human resources, organizational resources). Researchers (Liu Y et al, 2008) [9] suggested that effective coverage could reflect the quality of health interventions, and it aims at measuring the corresponding health improvement by health interventions. Hence, we designed the indicators for rural public health system coverage in three aspects, which were: 1) public health resource allocation (e.g. public health personnel); 2) public health interventions (e.g. safe drinking water); and 3) essential public health services [24]–[25]. A list of indicators for all the major interventions that targeted the most common public health issues and the health risk factors in China were made and validated by peer review. Indicators which are fundamental, suitable and sensitive to assess the public health interventions nationally are selected in the toolbox (Table 1).

**Table 1:** Indicators of rural public health system coverage and the associated measurement strategies.

Indicator	Intervention	Population in need	Definition of indicator	Year available
Safe drinking water	Access to safe drinking water	All households	Percentage of households with access to safe drinking water	2008–2010
Health record	Establish health record for each resident	All households	Percentage of residents who had been offered the health record	2008–2010
Children vaccination	Immunization	Preschool children (aged 0–6 years)	Percentage of children younger than 6 who were immunized with all kinds of vaccines that requested in the National Immunization Program^24^	2008–2010
Public health personnel	Deploy and develop public health staff	Public health staff who work in the county areas	Number of public health personnel who work in county health institutions (county hospitals, CDC and MCH Center), township hospitals and village clinics per 1000 local people	2008–2010
Health institutions accessibility	Reasonable regional distribution of health institutions	All households	The distance from rural residents' homes to the nearest health institutions (Units: km)	2010
Delivery	Hospital delivery	Women who gave birth in a specific year	Percentage of women who gave birth, deliver their baby in a hospital in the year before the survey	2008–2010
Maternal systematic management	Antenatal care and postnatal care	Expectant mothers and women who gave birth in a specific year	Percentage of women who gave birth in the year before the survey and received sufficient antenatal and postnatal visit by medical staff according to the National Basic Public Health Service Specifications	2008–2010
Hypertension treatment	Treatment and systematically management of hypertension patients	Hypertension patients	Percentage of hypertensive patients who reported having taken control measures and involved in the systematic management in the village before the survey	2008–2010
Diabetes treatment	Treatment and systematic management of diabetes patients	Diabetes patients	Percentage of diabetes patients who reported having taken control measures and involved in the systematic management in the village before the survey	2008–2010
Incidence of infectious diseases	Infectious disease prevention and control	All households	Percentage of per 100 thousand persons who had infectious diseases in the year before the survey	2008–2010

Second, the equality level of rural public health services among the typical population with different characteristics was assessed (Table 2). As children aged 0–6, adults aged above 65, hypertension patients, maternal women are the main consumers of essential public health services, they were considered as the target population. (A) Children were categorized into left-behind children and non-left-behind children groups because their guardians are responsible for their immunization and health exam. The left-behind children group was defined as children whose parents worked outside the county for more than 6 months in a year. (B) As researches (Lu et al, 2012; Tang et al, 2004) [26]–[27] showed that self-care compliance among hypertension patients is largely influenced by the economic conditions of their families, hypertension patients were categorized into two groups (low-income group vs. middle and high-income group) based on the rural per capita net income. (C) In postnatal care for maternal women, healthcare providers have the main responsibility to do the health check-ups for mothers and their babies. Thus, we divided maternal women into two groups, which were 1) living in suburban villages; and 2) living in general villages.

**Table 2:** Indicators of equality of public health services and associated measurement strategies.

Indicator	Population in need	Definition of indicator
Equality level of children with effective management	Children aged 0–6	OR of effective management* rate between left-behind children group and living-with-parents children group
Equality level of hypertension patients receiving follow-up regularly	Hypertension patients	OR of receiving follow-up regularly^#^ between the group of hypertension adults in low-income group and middle and high income group
Equality level of women with effective postnatal care	Women who gave birth in a specific year	OR of receiving postnatal care regularly^#^ between women living in suburban villages and whom in general villages

* Effective management means that a child has taken all immunization vaccination at his/her age and has undergone physical examination at least once in the recent year.

#People received sufficient follow-up visits according to the National Basic Public Health Service Specifications.

Third is the measure of rural public health services effectiveness. The main goal of China's public health system is to improve rural residents' health sustainability. It depends on the quality of public health services, awareness of health knowledge and development of public health system [28], [29]. Three sensitive indicators were selected for the measurement, which were: 1) hypertension control effectiveness; 2) satisfaction degree to the rural public health services; and 3) health knowledge awareness (Table 3).

**Table 3:** Indicators of effectiveness of public health services and associated measurement strategies.

Indicator	Intervention	Population in need	Definition of indicator
Hypertension control effectiveness	Effective treatment of hypertension	Hypertension patients	Percentage of hypertension patients who reported having taken control measures and whose blood pressure were normal in last three months before the survey
Satisfaction degree	Access to satisfactory health services	All households	Rural residents self-reported the satisfaction about the service delivery of the rural public health on a scale of one to ten (where ten is Perfect).
Health knowledge awareness	Health education and promotion	All households	Number of correct responses to ten questions which were selected from the Basic Knowledge and Skill of Chinese Citizens' Health Literacy^28^

### Study design and data sources

The data were collected from a cross-sectional survey conducted from July to November 2011. A multistage stratified random sampling method was used in this study. The provincial administrative regions of China were divided into four groups based on the human development index (HDI). [30] One province was randomly selected from each group as a sample, with the results of Zhejiang (region I), Henan (region II), Chongqing (region III) and Qinghai (region IV) were chosen for representing 4 development levels in China, and their rural per capita net income were RMB 11,302.5, RMB 5,523.73, RMB 5,276.66 and RMB 3,862.68 respectively. [31] In each province, 3 counties, 3 towns from each county and 3 villages from each town (1 suburban villages and 2 general villages) were selected following the sequence of district-block-residential area. Totally, 4 provinces, 12 counties, 36 townships, and 118 villages in the rural region were selected as the study areas. In each village, 8 hypertension patients, 8 children under 6 years old, 8 adults older than 65, and 3 women who gave birth in the past six months were randomly selected from the eligible candidates listed in health records. At last, 922 hypertension patients, 878 children, 848 elderly and 271 women responded to the study with response rates equal to 97.67%, 93.01%, 89.83% and 76·55% respectively. All participants were interviewed in person except the children's questionnaires were finished by their guardians. The School of Medicine and Health Management of Tongji Medical College and staff of the local health care institutions were recruited and trained as interviewers.

### Statistical analysis

Duplicate data entry method was adopted for entering all the data into the EpiData Info version 3.1 database and statistics program (Atlanta, Georgia, USA). Data entry screens were used to revise incorrect entries (e.g. logical errors, input errors). Statistical analysis was performed in SPSS statistics 12.0 (SPSS, Chicago, IL, USA).

Chi-square and Fisher's exact tests (whenever appropriate) were used to explore the regional differences of the variables. Odds ratio (OR) was adopted for measuring the equality level of rural public health services among different target groups. An OR ranging from 0.9 to 1.1 indicates that the public health services in the two groups are equal. A value of 1 represents perfect equality. ORs ranging from 0.7 to 0.8 or from 1.2 to 1.4 indicate relative equal. ORs ranging from 0.4 to 0.6 or from 1.5 to 2.9 indicate relatively unequal. ORs beyond 0.3 or 3.0 indicate totally unequal. All reported p-values are two-sided, and statistical significance level (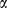) was set at 0.05. [32]

Questionnaire about participant's self-reported satisfaction about the rural public health service delivery was designed in a ten point Likert scale (ten represents perfect). On the other hand, the overall health knowledge score was determined based on the number of correct answers to ten health knowledge questions, which were randomly selected from the Basic Knowledge and Skill of Chinese Citizens' Health Literacy [28]. Incorrect, missing answers and the answer “don't know” were not counted.

## Results

### Socio-demographic characteristics of the participants

Hypertension patients were predominantly middle-aged or older persons and had a mean age of 64.31 years (SD = 11.40 years, range = 28–92 years) with the majority being female (54.6%). 50.98% of the hypertension patients were classified as low income, while more than 80% of them were below or at elementary educational level. The 271 mothers who gave birth in the past year were mainly aged 21–30 (71.96%), with mean age 27.54 years (SD = 6.95 years, range = 20–55 years). Most of them (81.92%) received middle school education or below. 29.27% of the 878 children were left-behind children, while most of them were raised by their grandparents. Adults older than 65 had a mean age of 72.50 years (SD = 6.14 years, range = 65–97 years), while 49.17% of them living with their descendants. More socio-demographic information can be found in Table 4 and Table 5.

**Table 4:** Socio-demographic characteristics of hypertension participants and women in rural China.

Hypertension patients			Women who gave birth in the past six months		
Items	Participants (n = 922)	Percent (%)	Items	Participants (n = 271)	Percent (%)
**Age**			**Age**		
<35	9	0.97	<21	13	4.80
35–44	41	4.45	21–25	114	42.07
45–54	117	12.69	26–30	81	29.89
55–64	238	25.81	31–35	39	14.39
65–74	349	37.85	36–40	12	4.43
>74	168	18.22	>40	12	4.43
**Gender**			**Gender**		
Male	419	45.40	Male	0	0.00
Female	503	54.60	Female	271	100.00
**Economic conditions**			**Economic conditions**		
Middle and high income	452	49.02	Middle and high income	149	54.98
Low income	470	50.98	Low income	122	45.02
**Education level**			**Education level**		
Less than 6 years elementary study	472	51.19	Less than 6 years elementary study	21	7.75
Elementary	285	30.91	Elementary	48	17.71
Middle school	116	12.58	Middle school	153	56.46
High school	35	3.80	High school	38	14.02
College and above	14	1.52	College and above	11	4.06

**Table 5:** Socio-demographic characteristics of children aged under six and adults older than 65 in rural China.

Children aged from 0 to 6			Adults older than 65		
Items	Participants (n = 878)	Percent (%)	Items	Participants (n = 848)	Percent (%)
**Age**			**Age**		
0–3	688	78.36	65–74	557	65.68
4–6	190	21.64	>74	291	34.32
**Gender**			**Gender**		
Male	476	54.21	Male	441	52.00
Female	402	45.79	Female	407	48.00
**Living conditions**			**Living conditions**		
Left-behind children	257	29.27	Living with descendants	417	49.17
Non-left-behind children	621	70.73	Living alone/living with husband or wife	431	50.83

### Performance of China's rural public health system

#### Coverage of China's rural public health system

Table 6 presents the performance of China's rural public health system coverage. It showed that the physical access to public health services generally increased from 2008 and 2010. China's rural public health system performed well in safe drinking water, children vaccinations and women hospital delivery, as indicated by the high coverage of these interventions. From 2008 to 2010, the overall coverage of the safe drinking water was increased from 91.81% to 94.44%, children vaccination and women delivery rates were maintained around 98%. Besides, the implement of essential public health services has a rapid increase during the three years, especially in the aspects of establishing health records for rural residents, maternal systematic management, systematic management of hypertension and diabetes. Health record establishing rate was increased dramatically by 25.48% per year, maternal systematic management rate had a 5.45% annual increase, and systematic management rate of hypertension and diabetes patients increased annually by nearly 15% on average. Moreover, the infectious-disease prevention and control had contributed to the 22.29 per hundred-thousand annual decrease of the incidence of infectious diseases in rural China. The accessibility of health institutions is moderate for rural residents with approximate 1 kilometer to the nearest health institution in 2010. The number of public health personnel per 1,000 rural population in rural China was still maintained at a very low level (1.59) in 2010, although there was an annual increase of 9.04% from 2008 to 2010.

**Table 6:** Performance indicators of rural public health system coverage in 2008, 2009 and 2010 by region.

Indicators of public health system coverage	Year	Rural	Region I	Region II	Region III	Region IV	Inter-region p-value
Safe drinking water*	2008	91.81(4.02)	97.34(1.11)	90.33(3.87)	91.71(4.93)	87.84(4.51)	>0.10
	2009	93.42(3.86)	98.22(0.71)	92.40(5.12)	94.14(4.47)	88.93(4.77)	>0.05
	2010	94.44(3.38)	98.93(0.62)	92.72(3.97)	94.86(4.59)	91.26(4.83)	>0.05
	Annual rate of change	1.32	0.80	1.20	1.58	1.71	−
Health record*	2008	30.32(20.1)	64.08(3.43)	0	15.92(2.51)	0	<0.0001
	2009	46.14(24.24)	78.42(2.50)	22.94(3.85)	33.18(2.95)	50.00(3.41)	<0.0001
	2010	71.62(11.59)	86.03(3.52)	63.33(4.51)	61.2(2.90)	75.91(2.95)	<0.0001
	Annual rate of change	25.48	7.61	31.67	28.02	37.96	−
Children vaccination*	2008	98.05(0.65)	98.01(0.90)	97.98(0.33)	98.90(1.12)	97.31(2.97)	>0.10
	2009	98.29(1.02)	98.87(0.65)	97.03(2.63)	99.33(0.40)	97.93(1.70)	>0.10
	2010	98.76(0.50)	98.47(0.77)	98.77(0.67)	99.45(0.58)	98.33(2.10)	>0.10
	Annual rate of change	0.35	0.23	0.395	0.275	0.51	−
Nearest distance to health institution (km)^#^	2010	1.09(0.23)	1.03(0.39)	1.07(0.38)	1.54(0.9)	1.19(0.6)	−
Number of public health personnel per 1,000 rural population#	2008	1.34(0.14)	1.43(0.28)	1.18(0.34)	1.27(0.13)	1.47(0.26)	−
	2009	1.48(0.11)	1.50(0.35)	1.35(0.36)	1.61(0.10)	1.45(0.20)	−
	2010	1.59(0.34)	1.28(0.15)	1.58(0.48)	2.08(0.14)	1.42(0.23)	−
	Annual rate of change	9.04	−4.89	15.72	27.98	−1.71	−
Delivery*	2008	97.89(2.21)	99.73(0.23)	99.62(0.60)	95.11(2.56)	97.11(2.73)	<0.05
	2009	98.44(2.06)	99.92(0.12)	99.86(0.19)	95.53(1.63)	98.46(2.03)	>0.05
	2010	98.53(2.00)	99.92(0.11)	99.97(0.05)	95.70(2.08)	98.51(2.18)	>0.05
	Annual rate of change	0.32	0.09	0.17	0.30	0.70	−
Maternal systematic management *	2008	76.39(11.98)	90.66(3.22)	61.39(3.54)	75.80(4.47)	77.72(2.81)	<0.0001
	2009	83.75(10.97)	94.43(1.14)	69.25(0.61)	89.47(1.41)	81.83(1.87)	<0.0001
	2010	87.30(8.70)	95.92(1.24)	75.61(1.67)	91.16(0.96)	86.50(4.86)	<0.0001
	Annual rate of change	5.45	2.63	7.11	7.68	4.39	−
Hypertension treatment*	2008	41.84(34.22)	71.06(1.62)	5.00(3.66)	70.89(5.10)	20.42(3.80)	<0.0001
	2009	62.30(21.27)	87.77(1.93)	56.67(4.67)	67.88(0.48)	36.89(0.33)	<0.0001
	2010	80.49(20.64)	97.27(2.62)	95.67(1.51)	75.80(0.66)	53.22(0.76)	<0.0001
	Annual rate of change	19.32	13.11	45.34	2.46	16.40	−
Diabetes treatment*	2008	55.36(17.78)	74.35(1.07)	66.18(1.02)	44.12(2.28)	36.79(2.46)	<0.0001
	2009	67.97(24.41)	92.38(5.59)	85.54(5.96)	46.00(4.22)	47.95(3.39)	<0.0001
	2010	76.39(15.59)	88.75(3.71)	90.08(5.18)	68.35(1.67)	58.38(1.25)	<0.0001
	Annual rate of change	10.80	7.20	11.95	12.12	10.80	−
Incidence of infectious diseases (per 100,000)	2008	396.05	478.65	370.57	448.17	286.80	<0.0001
	2009	397.24	386.75	385.77	467.74	348.7	>0.05
	2010	351.47	381.03	311.77	387.93	325.15	>0.10
	Annual rate of change	−22.29	−48.81	−29.4	−30.12	19.18	−

* Data are percentages (SD). # Data are numbers (SD).

On the other hand, remarkable inter-regional differences existed in rural public health system coverage. Except for the indicators of safe water and children vaccination, there were significant regional differences on establishing health records, women hospital delivery, hypertension treatment and infectious diseases incidence. In general, the coverage of rural public health system in region I was almost 10% higher than those in other regions. In 2008, the health record establishing rate of region I was 64.08% on average, whereas Region II and Region IV had no health record established. Although the two regions had a dramatic annual increasing rate of 31.67% and 37.96% respectively and the corresponding health record establishing rates was increased to 63.33% and 75.91% in 2010, the percentages were still lower than region I. Regarding the maternal systematic management, the highest increasing rate occurred in the Region III (91.16%) with an annual increase rate of 7.68%, but the rate was still slightly lower than Region I (95.52%) in 2010. Furthermore, Region II had a dramatic increase in hypertension treatment with an annual increase of 45.34%, which was a rapid development on chronic diseases' systematic management. We noticed that Region IV had the slowest progress on systematic management of hypertension and diabetes with the rate of systematic management equal to 53.22% and 58.38% in 2010 respectively, and the region had an annual increase of 19.18% on incidence of infectious diseases (Table 6).

#### Equality of accessing the public health services

Table 7,8,9 describes the equality of accessing the public health services in rural China for people in different communities. Generally, providing healthcare for hypertension people and postnatal care for maternal people were relatively equal in rural areas (OR ranging from 1.2 to 1.4). Low-income hypertension patients were less likely to receive regular healthcare follow-up comparing with middle and high income hypertension patients (OR = 1.2825). Besides, women living in suburban villages who are nearer to township hospitals were more likely to receive regular postal care than those living in ordinary villages (OR = 1.2491). For left-behind children, although they may receive less care from their parents, the health services they received was better than the living-with-parents children group (OR = 0.6242).

**Table 7:** Performance indicators of rural public health services' equality in 2010 by region (children).

Children	Non-effective management in ordinary children group*	Effective management in ordinary children group*	Ratio	Non-effective management in left-behind children group*	Effective management in left-behind children group*	Ratio	OR^#^
I	36 (26.87)	98 (73.13)	0.3673	17 (27.42)	45 (72.58)		
II	26 (12.81)	177 (87.19)	0.1469	14 (25.93)	40 (74.07)	0.3500	2.3827
III	76 (53.52)	66 (46.48)	1.1515	28 (37.33)	47 (62.67)	0.5957	0.5174
IV	49 (33.56)	97 (66.44)	0.5052	17 (25.76)	49 (74.24)	0.3469	0.6868
Rural	187(40.22)	278(59.78)	0.6727	76 (29.57)	181(70.43)	0.4199	0.6242

* Data are numbers (%).

# Positive results of OR are non-effective management. An OR ranging from 0.9 to 1.1 indicates that the public health services in the two groups are equal. A value of 1 represents perfect equality. ORs ranging from 0.7 to 0.8 or from 1.2 to 1.4 indicate relative equal. ORs ranging from 0.4 to 0.6 or from 1.5 to 2.9 indicate relatively unequal. ORs beyond 0.3 or 3.0 indicate totally unequal.

**Table 8:** Performance indicators of rural public health services' equality in 2010 by region (hypertension patients).

Hypertension patients	Not received healthcare regularly in middle and high income group*	Received healthcare regularly in middle and high income group*	Ratio	Not received healthcare regularly in low-income group*	Received healthcare regularly in low-income group*	Ratio	OR^#^
I	60 (50.42)	59 (49.58)	1.0169	40 (43.96)	51 (56.04)		
II	12 (15.79)	64 (84.21)	0.1875	29 (23.97)	92 (76.03)	0.3152	1.6812
III	31 (30.69)	70 (69.31)	0.4429	34 (50.00)	34 (50.00)	1.0000	2.2581
IV	13 (8.33)	143 (91.67)	0.0909	35 (18.42)	155 (81.58)	0.2258	2.4839
Rural	116(35.91)	207(64.09)	0.5604	138(41.82)	192(58.18)	0.7187	1.2825

* Data are numbers (%).

# Positive results of OR are not received healthcare regularly. An OR ranging from 0.9 to 1.1 indicates that the public health services in the two groups are equal. A value of 1 represents perfect equality. ORs ranging from 0.7 to 0.8 or from 1.2 to 1.4 indicate relative equal. ORs ranging from 0.4 to 0.6 or from 1.5 to 2.9 indicate relatively unequal. ORs beyond 0.3 or 3.0 indicate totally unequal.

**Table 9:** Performance indicators of rural public health services' equality in 2010 by region (women).

Women	Not received postnatal care in suburban village group*	Received postnatal care in suburban village group*	Ratio	Not received postnatal care in ordinary village group*	Received postnatal care in ordinary village group*	Ratio	OR^#^
I	12 (36.36)	21 (63.64)	0.5714	15 (38.46)	24 (61.54)		
II	11 (33.33)	22 (66.67)	0.5000	15 (31.91)	32 (68.09)	0.4688	0.9375
III	5 (27.78)	13 (72.22)	0.3846	19 (55.88)	15 (44.12)	1.2667	3.2933
IV	13 (39.39)	20 (60.61)	0.6500	13 (38.24)	21 (61.76)	0.6190	0.9524
Rural	41(35.04)	76(64.96)	0.5395	62(40.26)	92(59.74)	0.6739	1.2491

* Data are numbers (%).

# Positive results of OR are not received postnatal care regularly. An OR ranging from 0.9 to 1.1 indicates that the public health services in the two groups are equal. A value of 1 represents perfect equality. ORs ranging from 0.7 to 0.8 or from 1.2 to 1.4 indicate relative equal. ORs ranging from 0.4 to 0.6 or from 1.5 to 2.9 indicate relatively unequal. ORs beyond 0.3 or 3.0 indicate totally unequal.

In order to confirm the characteristics which could explain the differences of public health services' utilization among the target groups, multivariate logistic regression analysis was applied on each target group. Explanatory variables included gender, age, economic condition, education level, whether or not left-behind children, types of village living in (suburban, ordinary village), types of primary health institution participants receiving health care from (village clinic, township hospital) and ways of follow-up visits (telephone, home visit). The results showed that whether or not left-behind children (OR = 1.371, 95%CI = 1.021–1.840, p = 0.035) and types of primary health institution (OR = 1.649, 95%CI = 1.185–2.295, p = 0.003) were the significant predictors of children's health management; and types of village (OR = 0.469, 95%CI = 0.265–0.832, p = 0.009) and ways of follow-up (OR = 0.076, 95%CI = 0.037–0.155, p = 0.001) were the significant predictors of postnatal care for women who gave birth in a specific year. Besides, the significant predictors of follow-up healthcare regularly of hypertension people were economic level (OR = 0.728, 95%CI = 0.561–0.945, p = 0.017). The results showed that the variables of whether left-behind children or not, types of village and economic level were suitable for grouping the participants.

Participants living in different regions showed different trends in equality accessing to public health services. In the equality assessment of health management for children, it was equal between living-with-parents children group and left-behind children group in region I. However, there were two different situations in the other three regions. In region II, more left-behind children could not receive effective management with a rate of 25·.93%, whereas the corresponding figure for living-with-parents children group was just 12.81%. On the other hand, region III and IV showed a totally opposite situation with lower rate of non-effective management in left-behind children groups. In the second part, income-related inequality of healthcare delivery for hypertension patients in rural areas was obvious. And intra-regional inequalities in each region were also remarkable, especially in region III and IV with the ORs equal to 2.2581 and 2.4839 respectively. More hypertension patients with low income could not receive regular healthcare services from primary health institutions in low economic development regions. In the third part, women living in region I, II and IV could be totally equal in getting the postnatal healthcare, no matter how far she lives from the primary health institutions. However, distance-related inequality was significant in region III with OR equals to 3.2933, which suggested women who lived far away from the towns were less likely to acquire the standard postnatal care.

#### Effectiveness of rural public health services

Table 10 showed the performance of rural public health services' effectiveness according to the three aspects, which were: 1) effectiveness of public health services; 2) satisfaction towards public health services' delivery; and 3) health knowledge awareness. As a whole, nearly 40% of the hypertension patients had taken blood control measures and the blood pressure was normal in more than three months. Inter-regional differences existed in rural areas with the highest effectiveness rate (52.67%) in region III, and the lowest in region IV (29.87%). Meanwhile, the hypertension treatment rate of region IV was just 53.22% in 2010 (Table 6) which suggested only 15% of the hypertension patients living in region IV had taken blood control measures and was able to keep it within the normal range.

**Table 10:** Performance indicators of rural public health services' effectiveness in 2010 by region.

Region	Hypertension control effectiveness (%)	SD	Satisfaction degree	SD	Health knowledge awareness	SD
I	45.90	19.94	7.93	1.17	5.84	2.20
II	42.37	11.29	8.99	1.52	5.13	2.29
III	52.67	11.83	7.50	1.19	3.55	1.87
IV	29.87	16.87	6.26	3.45	4.10	2.14
Rural	39.70	18.95	7.53	2.52	4.64	2.31

We also noted that the residents in more developed regions were more satisfied with the public health services in their region. As a whole, the rural residents have a degree of relative satisfaction to rural public health services' delivery (7.53 out of ten). The highest degree occurred in region II (8.99), whereas region IV had the lowest degree (6.26). Comparing with hypertension patients (satisfaction degree: 8.37) and women who gave birth in last year (satisfaction degree: 7.85), adults aged older than 65 were less satisfied (6.82) with the villages' public health services for them.

Table 10 also presented the health knowledge awareness of rural participants in the four regions. The mean number of correct items of all the participants was 4.64 out of ten and that of hypertension patients, women and old people were 4.67, 4.89 and 4.57 respectively. Obviously, inter-regional difference existed in health knowledge awareness. Participants in region I correctly answered six questions on average, while that of region III and IV was just four. It is worth to notice that the women and old people in region III had the lowest number of correct items which was just three, while those who living in region I correctly answered on average.

## Discussion

The comprehensive measurement of China's rural public health system performance has been considered as having only one major variable: the public healthcare delivery which addressed the basic questions of whether rural residents could receive basic public health services and whether they could receive those services equally. The three years data and the nationally representative survey indicated positive trends of access and utilization of public health services in rural China. The trends suggested that China's rural public health system performance did well in some aspects during the three years, such as safe drinking water, establishing health records, children immunization, hospital delivery, and infectious-disease prevention and control. However, low performance was observed in equality and effectiveness of children management (i.e. vaccination and annual physical check) and people with chronic diseases (i.e. hypertension and diabetes).

Implementation of the health reform since 2009 has headed the China's public health system to the right direction and promoted the improvement of rural public health system performance. Nearly nationwide national insurance coverage was achieved within a short time is remarkable [17]. Health record establishing rate in rural areas was raised 41.30%, and dramatic increase occurred in the lowest HDI regions (i.e. Region IV) with 0 in 2008 and rising to 75.91% in 2010. Besides, rural residents could access to the primary healthcare in around 1 kilometer, this should attribute to the policy that each village in China should establish one standard village clinic. Because of the implementation of basic public health package, more attention has been paid by the primary health institutions on public health. The delivery of basic public health services has become the major duty of primary healthcare providers. Although the creditable increase occurred in the coverage of public health services, we found that unsatisfied quality of rural public health services affected the performance of rural public health system. For example, nearly 80% of the hypertension patients had taken systematic management in 2010, but only 61% of them could receive follow-up regularly and just 39.70% of them were able to keep their blood pressure within the normal range. Moreover, 87.3% of the women were managed systematically in 2010, but only near 61% of them could obtain the postnatal cares on schedule. Therefore, how to ensure and enhance the quality of public health services is the key problem that the reform has to face.

China has recognized the major deficiencies in its health-care system, which are inequities and shortages of human resources. [33] Although the gap between regions was gradually narrowed in the past three years, inter- and intra-regional inequalities still exist in the rural public health system. The multiple logistic regression analysis presented that household income, distance from home to primary health institutions were the significant predictors of provincial public health system equality. Theoretically, basic public health services should be provided proactively by health providers. However, due to the shortage of human resources, the services of follow-up were normally provided though rural residents visiting the primary health institutions instead of home visit by health providers. The distance and travel cost could be the obstacles for rural people getting the public health services. Although the government has identified training new physicians as a top priority and plans to train 300,000 new physicians over the next ten years, expanding the team of public healthcare providers in rural areas has been difficult. [31], [34] This research showed that there was just 1.59 (per 1,000 rural population) public health providers in rural China in 2010 including professional and part-time providers. Moreover, only 0.0596 (per 1,000 rural population) professional public health providers were having the responsibility to provide basic public health services for the residents in rural China. Insufficient human resource caused the problem of inequality and inadequate of public health services [35]. The human resource related inequality of rural public health services is another problem that the reform has to face.

With respect to the public health services' delivery for children, one remarkable result showed that the left-behind children in western China (region III and IV) had a higher rate of receiving the effective management than that of living-with-parents children, although it was much lower than region I and II. As the health condition of left-behind children has gradually been widely noted [36], local policies for protecting and promoting the health-related quality of life of left-behind children have been formulated during these years. In western China, health management for left-behind children has become one of the most important government issues, and village clinics and village committee have taken the responsibility to provide safeguard for them. Although the health condition of vulnerable groups (e.g. children and elderly) in rural China is gradually concerned by the Government at all levels, more efforts and attentions shall be paid to them for achieving the goal of population-wide health improvement and the reduction of health inequalities.

More than 80% of participants in our study had inadequate heath knowledge, including the highly educated rural participants who had high school education or higher. Nearly 95% hypertension participants and 82% women just had less than 9 years' basic education. Low-literacy situation of rural residents is one of the obstacles of effective implementation of health education and promotion [37]–[38]. Meanwhile, it is reported that nearly 90% of village physicians are below vocational educational level, and one third of them practice without a degree [4]. Most of them lack knowledge about disease prevention and control, so it is difficult for them to provide health knowledge to local residents. These could result in the low performance of health education and promotion in rural China.

## Limitations

Our analysis has several limitations, which need to be considered when interpreting the research findings. Our data of health knowledge and satisfaction towards public health services' delivery were based on self-report answers, which may be subject to response bias. Another potential limitation of our research is the use of closed-ended questions (i.e. true/false/don't know) in the health knowledge test, which allowed participants to guess the correct answer.

## Conclusions

Implementation of the health reform since 2009 and more attention paying on the rural public health system has headed the rural public health system to the right direction and promoted the performance improvement. Nearly nationwide safe drinking water, health record and children vaccination coverage was achieved within a short time. At the same time, the problems of poor quality and equality of public health services are also highlighted. China's rural public health system should be intensively improved in the aspects of quality of public health services and the equality among regions. Proper implementation of public health policies requires independent scientific monitoring and evaluation to enable midcourse correction and determine the weaknesses and the causes.
